# Supplementary material for: Causal interplay between lactose intolerance and gut microbiota: a combined bidirectional Mendelian randomization and in vivo validation study
Source: Front Nutr. 2026 Jun 1;13:1803337. doi: 10.3389/fnut.2026.1803337 (PMC13265576; doi:10.3389/fnut.2026.1803337)
Supplement: Supplementary file 1 [file Data_Sheet_1.zip › supplementary materials/Forward/forest plot/ebi-a-GCST90027713.finngen_R12_E4_LACTONAS.pdf]

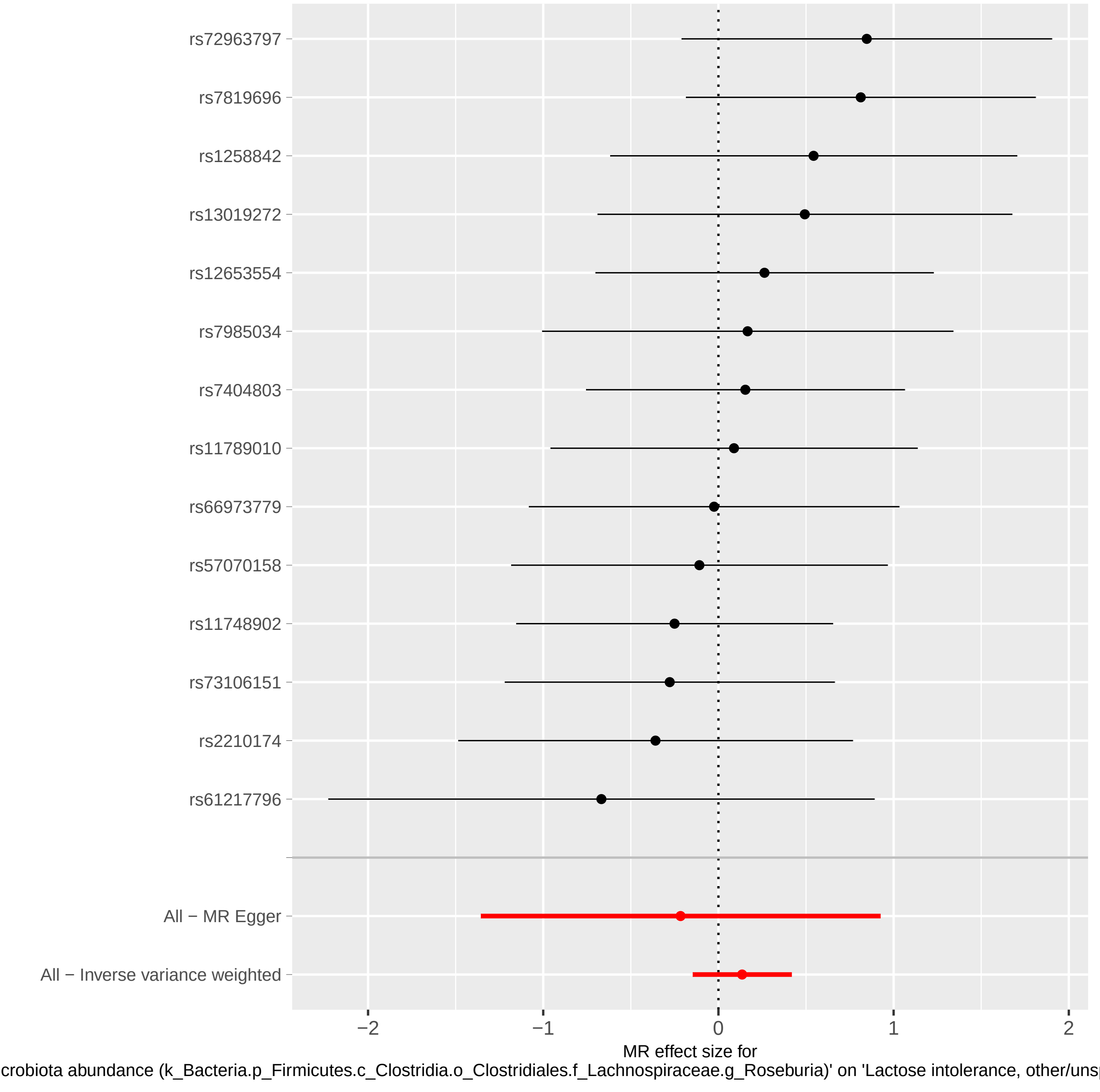

rs72963797
rs7819696
rs1258842
rs13019272
rs12653554
rs7985034
rs7404803
rs11789010
rs66973779
rs57070158
rs11748902
rs73106151
rs2210174
rs61217796
All − MR Egger
All − Inverse variance weighted
−2
−1
0
1
2
MR effect size for
crobiota abundance (k_Bacteria.p_Firmicutes.c_Clostridia.o_Clostridiales.f_Lachnospiraceae.g_Roseburia)' on 'Lactose intolerance, other/uns
